# Supplementary material for: Effectiveness and Safety of Ayurvedic Medicines in Type 2 Diabetes Mellitus Management: A Systematic Review and Meta-Analysis
Source: Front Pharmacol. 2022 Jun 8;13:821810. doi: 10.3389/fphar.2022.821810 (PMC9213670; doi:10.3389/fphar.2022.821810)
Supplement: Supplementary file 1 [file DataSheet1.zip › Supplementary Material/Forest and Funnel Plots/Gynostemma pentaphyllum/BMI.pdf]

Gynostemma pentaphyllum - BMI

	Intervention			Comparator			Mean Diff.	Weight
Study	N	Mean	SD	N	Mean	SD	with 95% CI	(%)
Huyen 2010	12	23.70	2.00	12	22.70	2.40	1.00 [-0.77, 2.77]	69.99
Huyen 2012	12	21.90	2.70	13	22.30	4.00	-0.40 [-3.10, 2.30]	30.01
**Overall**							0.58 [-0.90, 2.06]	

Heterogeneity: τ^2 = 0.00, I^2 = 0.00%, H^2 = 1.00

Test of $\theta_i = \theta_j$: Q(1) = 0.72, p = 0.40

Test of θ = 0: z = 0.77, p = 0.44

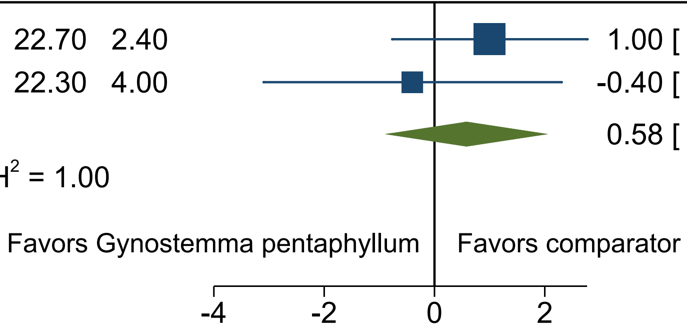

Random-effects REML model
